# Characterization of fecal microbiota in cervical cancer patients associated with tumor stage and prognosis

**DOI:** 10.3389/fcimb.2023.1145950

**Published:** 2023-02-23

**Authors:** Lei Chang, Luojie Qiu, Ningjing Lei, Junying Zhou, Ruixia Guo, Feng Gao, Shiliang Dong, Mengyu Chen, Fengling Wu, Bo Qin

**Affiliations:** ^1^ Department of Obstetrics and Gynecology, The First Affiliated Hospital of Zhengzhou University, Zhengzhou University, Zhengzhou, Henan, China; ^2^ School of Basic Medical Sciences, Zhengzhou University, Zhengzhou, Henan, China; ^3^ Department of Neuroimmunology, Henan Institute of Medical and Pharmaceutical Sciences, Zhengzhou University, Zhengzhou, Henan, China; ^4^ Henan Engineering Technology Research Center for Accurate Diagnosis Neuroimmunity, Zhengzhou, Henan, China; ^5^ Department of Radiation Oncology, The First Affiliated Hospital of Zhengzhou University, Zhengzhou, Henan, China; ^6^ Translational Medical Center, The First Affiliated Hospital of Zhengzhou University, Zhengzhou, Henan, China

**Keywords:** cervical cancer, fecal microbiota, 16S rRNA sequencing, metagenomic next-generation sequencing, *Ruminococcus 2*

## Abstract

Cervical cancer (CC) is the fourth most frequent malignancy among women worldwide, and its prevention and treatment are evolving rapidly. The gut microbiota has been reported to play a crucial role both in the preservation of homeostasis and the development of cervical cancer. In this study, we collected fecal samples to investigate the microbial signatures in cervical cancer patients compared with healthy controls using 16S rRNA sequencing analysis and metagenomic next-generation sequencing (mNGS) testing. Our findings demonstrated a substantial difference in the gut microbiota composition of cervical cancer patients and healthy controls. The disease and stage were most significantly negatively correlated with *Ruminococcus 2*, which might be considered a potential clinically relevant biomarker. Functions of differential microbiomes were also analyzed, indicating significant differences in metabolisms and biosynthesis between the two groups. These findings demonstrate that patients with cervical cancer have certain species of gut microbiota that are exclusive to them and particular species have the potential to be used in the prognosis of cervical cancer.

## 1 Introduction

Cervical cancer poses a major threat to the health of women globally with around 520,000 new cases and 270,000 deaths each year (Siegel et al., 2020). Although the human papillomavirus (HPV) infection is a significant risk factor for cervical cancer, most HPV infections are temporary, with just 10-15% of high-risk infections remaining and 85-90% of high-risk infections spontaneously resolving. The persisting HPV infection causes precancerous cervical intraepithelial neoplasia (CIN), which further develops into invasive cervical cancer (ICC) (Shulzhenko et al., 2014). Understanding the processes involved in the tumorigenesis of cervical cancer is still very important. In addition, the early diagnosis and prognosis of cervical cancer provide important information for clinical application.

The human body is colonized by a diverse range of commensal and pathogenic microbial communities, including protists, archaea, bacteria, fungi, and viruses (Gilbert et al., 2018). Recent studies have found that gut microbiota is linked to the occurrence and progression of cancerous diseases. For example, common P53 mutations exert carcinogenic effects in the case of gallic acid produced by microorganisms (Kadosh et al., 2020). In the meanwhile, the commensal gut microbiome may functionally interact with the host’s genome to provide protective effects by secreting bioactive compounds. In some cases, the secretion and metabolism of estrogen are greatly affected by gut microbial functions (Plottel and Blaser, 2011; Flores et al., 2012). This results in an estrogen-mediated gut-vagina axis (Baker et al., 2017). The interaction between the gut and the distal vaginal mucosa involves certain microorganisms that metabolize estrogen. The collection of this kind of microbiota is known as estrobolome (Plottel and Blaser, 2011). The alterations in the diversity of the gut microbiota may affect how estrogen is metabolized. And estrogen mediates the production and secretion of glycogen in the vaginal epithelium, resulting in high levels of free glycogen that affect the composition of the vaginal microbiota (Mirmonsef et al., 2016). Vaginal flora imbalance is closely related to HPV infection and cervical intraepithelial lesions. At the same time, estrogen itself can act on HPV response elements to change viral gene expression, as well as accelerate the process of HPV infection (Chen et al., 2021; Wang et al., 2021; Zhang et al., 2021b). And it can also play an immunomodulatory role in the tumor microenvironment to promote the occurrence and development of HPV-positive cervical cancer. Thus, the constituents and effects of microbiomes should be investigated to study the related mechanism in certain diseases (Gagnière et al., 2016; Liu et al., 2018).

In cervical cancer, a study compared the gut microbiome composition of five healthy controls and eight patients to establish a strong association between the gut microbiome and cervical cancer (Wang et al., 2019). They found that the distribution of gut microbiota differed between cervical cancer patients and healthy people, and the gut microbiome may provide potential diagnostic biomarkers for cervical cancer, including *Parabacteroides*, *Escherichia Shigella*, and *Roseburia*. Another study also observed different genres in fecal microbiota dynamics between healthy women and patients with early cervical cancer (Kang et al., 2020). They found that in the cancer group, Proteobacteria were significantly more abundant, including *Escherichia*, *Coli-Shigella*, *Rossella*, *Pseudomonas*, *Clostridium pilonicum*, *Pilospirillaceae*, *Yersinia*, and *Vibrio Succinosus*. Although these studies provide some proof that gut microbiota is different between cervical cancer patients and healthy controls, functions and mechanisms have not been further explored yet.

In this study, we collected fecal samples from 13 patients with cervical cancer and 10 healthy controls. The clinical characteristics of all individuals were analyzed. 16S rRNA sequencing analysis and mNGS testing were used to identify the characteristics of gut microbiota to identify and compare the microbiome. Functions and related mechanisms were further conducted. Our results showed that *Ruminococcus 2* was the most significantly different microorganism, which might be considered a potential clinically relevant biomarker.

## 2 Methods

### 2.1 Research overview

In this study, patients with cervical cancer and healthy controls who were admitted to the hospital from February 2022 to June 2022 were collected. The choice was taken and the informed permission was signed by the patients and their families. Following admission, the patient’s conditions and the progress of the study were fully disclosed to them as well as to their families. The Zhengzhou University First Affiliated Hospital Ethics Committee has authorized this work (2022-KY-1341-002). The eligibility criteria are as follows: (1) Age: 18-72 years old; (2) All patients with cervical cancer were pathologically diagnosed; All healthy controls underwent detailed CT, MRI, and other imaging examinations. (3) All patients had not received pelvic and systemic concurrent chemoradiotherapy before admission, and the main organ functions were normal. (4) None of the participants had antibiotics administration 3 months before samples collection. And the two groups were matched by age and body mass index (BMI).

### 2.2 Collection of stool samples

Each subject gave a sample of their most recent tail feces between 06:00 and 10:00 AM. Samples were separated into 200mg aliquots and immediately kept at - 80°C after being inactivated at 70°C for 1 hour. Samples that were left at room temperature for more than two hours were discarded.

### 2.3 16S rRNA sequencing analysis and data processing

NovaSeq 6000 SP Reagent Kit V1.5 (Illumina, USA) was used for PE250 sequencing and QIIME2 was used for preliminary analysis of sequencing data to obtain microbial annotation and other data, and R software (version 4.2.2) was used for statistical analysis.

### 2.4 Operational taxon clustering and taxonomic annotation

The same number of reads were randomly selected from all samples and classified into operational taxonomic units (OTUs) by the UPARSE pipeline. We gathered every OTU from every sample used in the discovery, validation, and independence phases. The identity threshold was set at 0.97. We carried out taxonomic studies and further investigations of microbial diversity.

### 2.5 Library construction

Covaris (Woburn, Massachusetts, USA) randomly fragmented 1 μg of genomic DNA. Magnetic beads were used to filter the DNA fragments to an average size of 200–400 bp. The libraries were then built using end-repair, A-tailing, and adapter ligation. Then it was put through a PE100 mode sequence on the BGISEQ-500 platform.

### 2.6 Statistical analysis

The Wilcoxon rank-sum test, the Kruskal-Wallis test, and Fisher’s exact test were used to assess differences between the two groups for non-normal continuous variables and categorical variables, respectively. Statistical analyses were performed using SPSS V.20.0 for Windows (Illinois, USA). The within-sample and between-sample diversity are represented, respectively, by the α and β diversity indices. In the quantitative insights into microbial ecology (QIIME, ver.1.9.1), α-diversity indices were calculated. The amount of differences between different samples was then calculated for the β -diversity analysis using Principal coordinates analysis (PCoA). The research was then aided by MetagenomeSeq analysis, which evaluated the effects of species abundance using linear discriminant analysis (LDA) effect size (LEfSe). Functional predictions of microbial communities were made using the Phylogenetic Investigation of Communities by Reconstruction of Unobserved States (PICRUSt) program. The extent to which environmental factors influence gut microbial changes were analyzed using CCA analysis. In the illustration, * *p* < 0.05, ** *p* < 0.01, and *** *p* < 0.001 was defined as statistically significant. In the principal coordinates analysis, aa=*p*<0.05, ab= *p*>0.05. Differences with *p*-values of < 0.05 (two-sided) were considered to indicate statistical significance.

## 3 Results

### 3.1 Clinical characteristics of the recruited subjects

**Figure 1:**
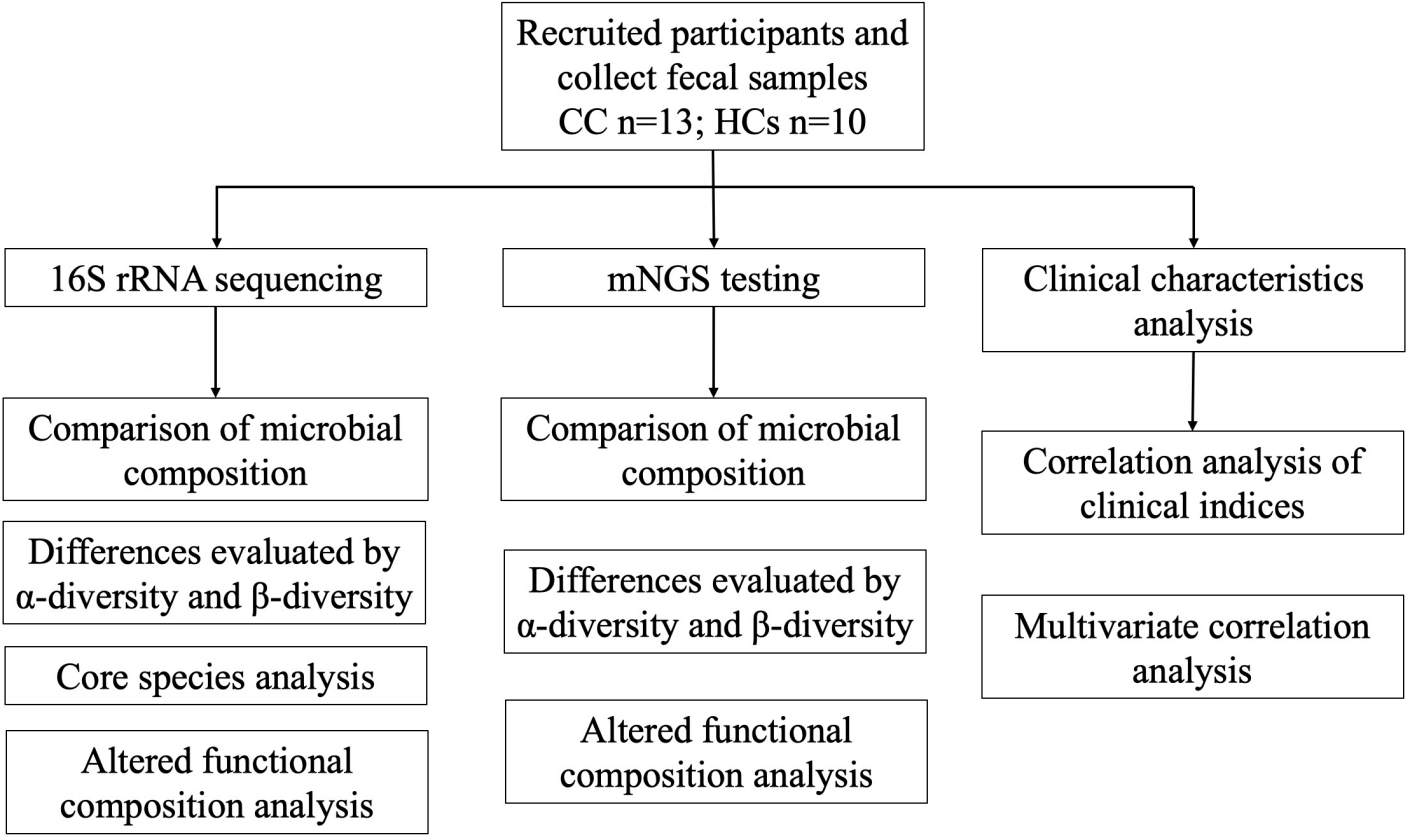
Flow chart of the study design. A total of 23 fecal samples were collected, including 13 cervical cancer patients and 10 healthy controls. Analysis in this study is summarized.

A total of 13 patients with cervical cancer (CC) and 10 healthy controls (HCs) were recruited in this study, and the experimental flow chart of this study is summarized (**Figure 1**). CC group was pathologically identified and new to cervical cancer. To confirm cervical cancer samples of histological kinds, pathological biopsies were performed on all cervical cancer patients. Age and BMI did not significantly differ between the two groups. The demographic and clinical traits of the CC group and HCs group are displayed in **Table 1**.

**Table 1:** Demographic characteristics of the study participants.

Characteristic	Overall, N = 23	CC, N = 13	HCs, N = 10	*p-value*
Patient Age, Mean	47.09 ± 9.17	48.92 ± 10.81	44.7 ± 6.22	0.284
BMI, Mean	25.11 ± 4.51	24.2 ± 3.19	26.29 ± 5.78	0.282
Gender, n (%)				
Female	23 (100%)	13 (56.4%)	10 (43.6%)	
Stage, n (%)				
I	6 (26.0%)	6 (46.2%)		
II	3 (13.0%)	3 (23.1%)	NA	
III	4 (17.4%)	4 (30.8%)		
Differentiation, n (%)				
High	5 (21.6%)	5 (38.5%)		
In	4 (17.4%)	4 (30.8%)	NA	
Low	4 (17.4%)	4 (30.8%)		
Tumor diameter, n (%)				
≦ 4 cm	9 (39.0%)	9 (69.2%)		
> 4 cm	4 (17.4%)	4 (30.8%)	NA	
Vaginal infiltration, n (%)				
No	10 (43.4%)	10 (76.9%)		
Yes	3 (13.0%)	3 (23.1%)	NA	

### 3.2 Gut microbial distribution and diversity between patients with cervical cancer and healthy controls

**Figure 2:**
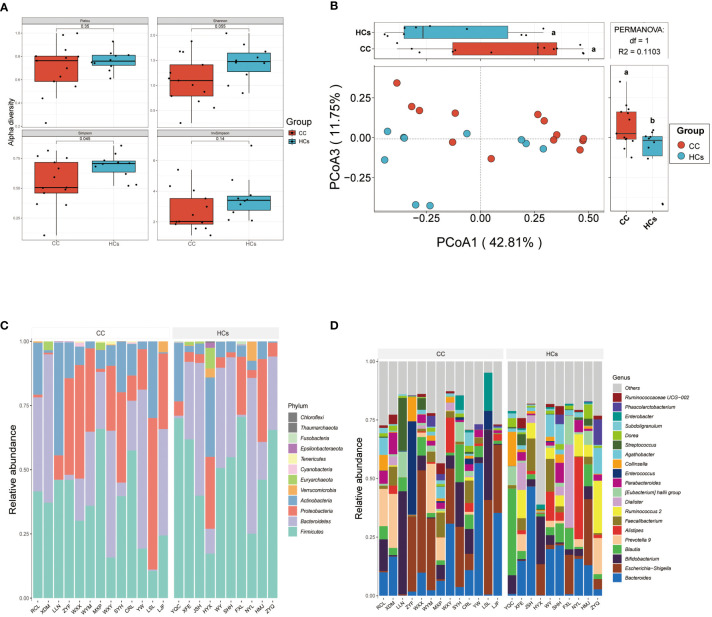
Gut microbial diversity differs in patients with cervical cancer compared to healthy controls. **(A)** α-diversity analysis (Pielou, Shannon, Simpson, and InvSimpson index). **(B)** PCoA analysis based on Pearson Distance and Bray-Curtis distance matrices. Each sphere represents one sample. Samples are separated into two groups. **(C)** The two groups’ average compositions and relative abundance at the phylum level. **(D)** The two groups’ average compositions and relative abundance at the genus level. aa *p*>0.05. ab *p*<0.05.

Differences in the gut microbiota between CC and HCs groups were evaluated by the α-diversity and β-diversity. α-diversity is commonly used to measure the richness of species in the ecology of a community and is a comprehensive indicator of species richness and evenness. While β-diversity refers to the species divergence between different environmental communities (Jost, 2007). β-diversity together with α-diversity makes up the overall diversity. The statistical difference in the α-diversity index in the two groups was not significant (**Figure 2A**). However, there was a tendency for microbial community diversity in the CC group to be lower than that in the HCs group. The average microbial composition diversity of the two groups was compared using the principal coordinates analysis (PCoA) to ascertain β-diversity. The Pearson Distance and Bray-Curtis Distance showed significant differences (aa=*p*>0.05, ab= *p*<0.05) in microbial communities between the CC group and the HCs group (**Figure 2B**). The fecal microbial composition was dominated by *Firmicutes*, *Bacteroidetes*, *Proteobacteria*, and *Actinobacteria* at the phylum level (**Figure 2C**). The genus level (**Figure 2D**) was dominated by *Bifidobacterium*, *Escherichia Shigella*, *Bacteroides*, and *Blautia*. There was no significant difference in the main flora of gut microflora between the CC group and HCs group at the phylum and genus levels, but there was a difference in the composition ratio of gut microflora. Although α-diversity did not show the difference in the abundance of gut flora, β-diversity indicated the difference between the CC group and HCs group. Combined with the differences at the phylum and genus levels, we observed differences mainly reflected in the species level of gut flora. Species diversity confirmed that gut microbial communities were different between the CC group and the HCs group.

### 3.3 Differential gut microbiota in cervical cancer patients

**Figure 3:**
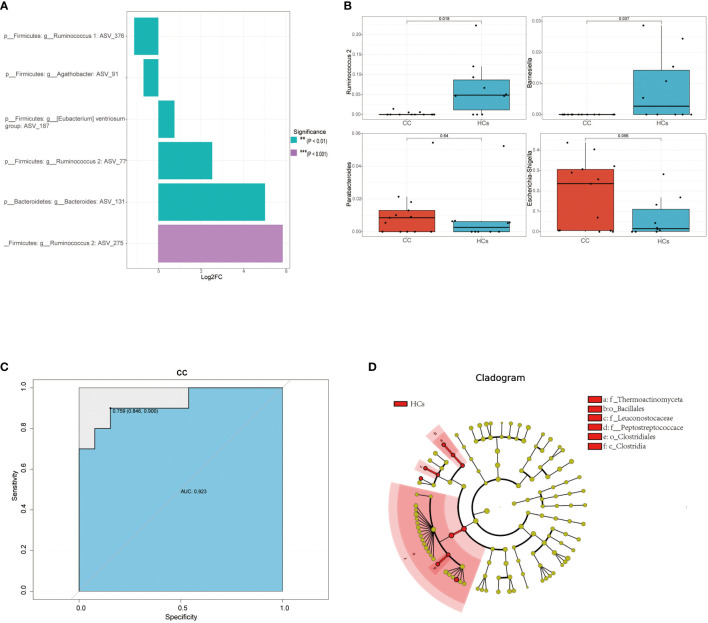
Differential gut microbiota in cervical cancer patients. **(A)** Linear discriminant analysis effect values species bar graph based on MetagenomeSeq analysis. **(B)** Core microorganisms based on random forest analysis (min_relative = 0.001, min_ratio = 0.7, p<0.05 for significant differences). **(C)** achieving an AUC value of 0.923 **(D)** Taxonomic cladogram from LEfSe, depicting the taxonomic association between the fecal microbiome communities from CC and HCs group. **p<0.01, ***p<0.001. AUC, area under the curve; CC, cervical cancer; HCs, healthy controls.

Based on 16s rRNA sequencing analysis, we next used the MetagenomeSeq analysis to avoid the influence of the Rarefaction process on the accuracy of the results to further explore differences between the CC group and the HCs group. In the MetagenomeSeq analysis, we found that *Ruminococcus 1*, *Agathobacter*, *Ventriosum group*, *Ruminococcus 2*, and *Bacteroides* were all differential (*p*<0.01). Among them, *Ruminococcus 2* showed the most significant difference (*p*<0.001) (**Figure 3A**). We then used Leave-one-out cross-validation to build a random forest classification model (*p*<0.05). Random forest analysis and ROC of core species were performed in randomly generated decision tree fitting input samples, which first filtered species level data and selected core microorganisms for analysis (min relative = 0.001, min ratio = 0.7, *p*<0.05 for significant differences), and found significant differences for *Ruminococcus 2* and *Barnesiella* (**Figure 3B**). The effect of this classification model was assessed using the receiver operating characteristic (ROC) curve with the area under the ROC curve (AUC) as 0.92 (**Figure 3C**). It indicated that low *Ruminococcus 2* in the gut flora family is most closely related to cervical cancer. In addition, LEfSe also found that microbes were the major contributor to sample differences (**Figure 3D**, LAD score > 2, *p* < 0.05). However, in our LEfSe analysis, all differential species were more abundant in the HCs group. Specific species have been identified in our analysis, which could be further studied for their association with cervical cancer progression. Among them, *Ruminococcus 2* might be a potential marker in cervical cancer patients.

### 3.4 Altered functional composition of gut flora in patients with cervical cancer

**Figure 4:**
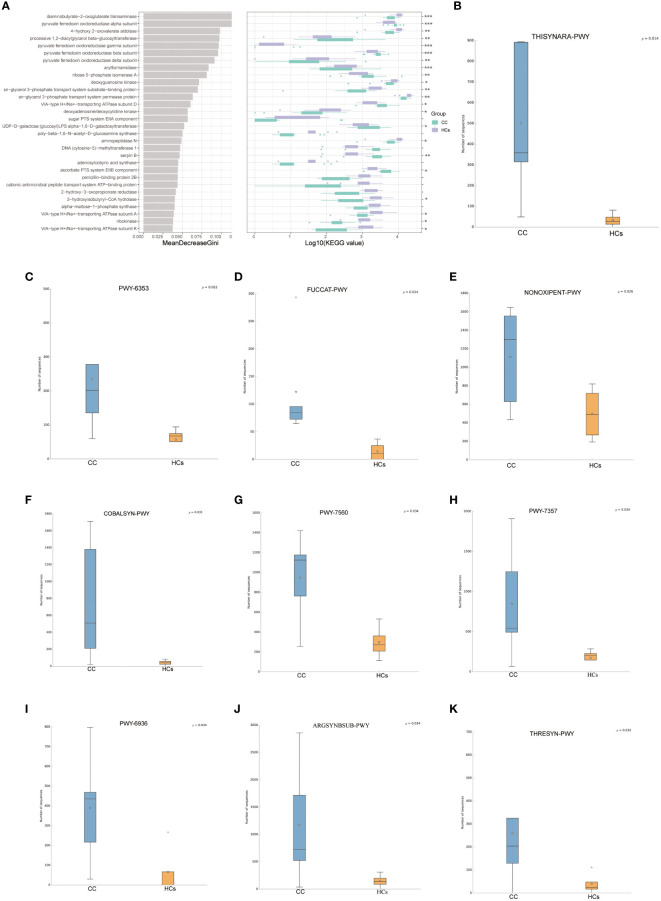
Predicted functionality of the fecal microbiota. **(A)** 30 enriched pathways with the most significant differences between the CC and HCs groups were identified based on KEGG by 16s rRNA sequencing analysis. **(B–K)** The top 10 metabolic pathways with differential abundance by mNGS testing.

To study the altered gut microbial function in CC patients, PICRUSt2 was used to annotate the OUT sequence and predict the function of 16S rRNA sequencing analysis. Through the combination of KEGG and other databases, gene annotation and biological interpretation were carried out. We detected the microbial functional abundance of the gut flora in the CC group and the HCs group, and the functions of the differentially expressed genes were mainly concentrated in REDOX reaction, biosynthesis of other secondary metabolites, amino acid transport, and metabolism (*p*<0.05, **Figure 4A**). To validate functional alterations in the gut microbiome, mNGS testing was applied for microbial function analysis. The fecal microbial samples from 9 participants closest to the test date (5 from the CC group and 4 from the HCs group) were selected for mNGS testing. We conducted the functional difference analysis of mNGS testing and found 41 significantly different results. The top 10 metabolic pathways with differential abundance were selected for distribution analysis (**Figures 4B–K**, and it was found that the abundance of 10 differential metabolic pathways was increased in the tumor group, including the superpathway of thiamine diphosphate biosynthesis III (THISYNARA-PWY), purine nucleotides degradation II (PWY-6353), fucose degradation (FUCCAT-PWY), superpathway of adenosylcobalamin salvage from cobinamide I (COBALSYN-PWY), methylerythritol phosphate pathway II (PWY-7560), thiamine phosphate formation from pyrithiamine and oxythiamine (PWY-7357), seleno-amino acid biosynthesis (PWY-6936), L-arginine biosynthesis I (ARGSYN-PWY), and superpathway of L-threonine biosynthesis (THRESYN-PWY). These results indicated significant differences in microbiota functions between the CC and HCs groups mainly falling in the production of precursor metabolites and energy, as well as in the biosynthesis of amino acids.

### 3.5 Relationship between gut flora and clinical indices

**Figure 5:**
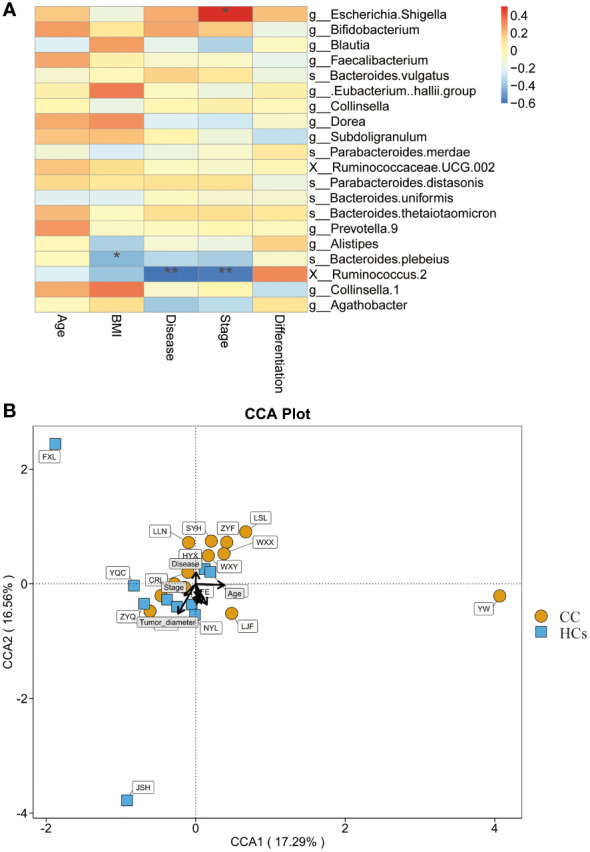
Relationship between gut flora and clinical indices. **(A)** Heatmap shows the correlation between clinical indices and gut flora. **(B)** CCA shows the correlation between clinical indices and microbial abundance. *p<0.05, **p<0.01. CCA, canonical correspondence analysis.

We also evaluated the correlation between clinical indices and gut flora. BMI, disease, and stage were found to be strongly correlated with the abundance of bacterial genera in the CC group (**Figure 5A**). BMI was negatively correlated with *Bacteroides Plebeius* (*p*<0.05). Disease and stage were significantly negatively correlated with *Ruminococcus 2* (*p*<0.01). And stage showed a positive correlation with *Escherichia Shigella* (*p*<0.05). In this study, distinct points were used to represent various samples while also including clinical markers and microbiological abundance for analysis. The length of the arrow, which indicated the intensity of each environmental factor’s influence on community transformation, reflected the extent of the environmental factor’s influence. The arrows from the origin represented various environmental factors. The correlation between the patient factor and the coordinate axis is indicated by the angle between the arrow and the axis, and the smaller the angle, the stronger the correlation. We calculated that Tumor-diameter (*p*=0.014) was associated with microbial abundance, but the magnitude of the correlation could not be determined (**Figure 5B**). These data further indicated that *Ruminococcus 2* was associated with cervical cancer disease and stage, making it a promising marker.

### 3.6 The mNGS testing of gut microbiota in patients with cervical cancer

**Figure 6:**
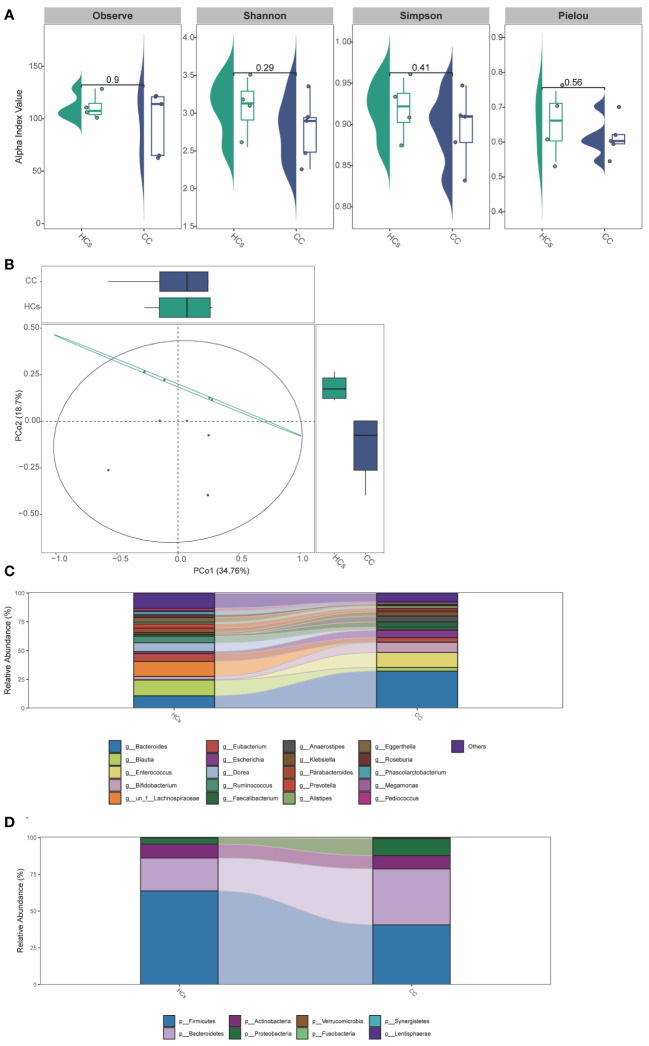
The mNGS testing of gut microbiota in patients with cervical cancer. **(A)** α-diversity analysis (Observe, Shannon, Simpson, and Pielou index). **(B)** PCoA analysis based on Pearson Distance and Bray-Curtis distance matrices. Each sphere represents one sample. Samples are separated into two groups. **(C)** The two groups’ average compositions and relative abundance at the genus level. **(D)** The two groups’ average compositions and relative abundance at the species level.

In addition to the functional tests described above, we validated the 16S rRNA sequencing analysis results with mNGS testing. The results suggested no significant difference in α-diversity between the two groups (**Figure 6A**). The difference in β-diversity between the two groups was along the second axis of the Pearson and Bray-Curtis distances, explaining 18.7% of the total variation (**Figure 6B**). The abundance of *Bacteroides* was significantly increased at the genus level (**Figure 6C**). At the species level (**Figure 6D**), the abundance of *Firmicutes* and *Bacteroidetes* was significantly reduced. The above results were consistent with 16S rRNA sequencing analysis between CC and HCs groups.

## 4 Discussion

In recent years, many studies have shown that gut microbiome dysregulation is increasingly associated with the development of tumors near or far from the gut tract (Xue et al., 2018; Cheng et al., 2020; Yang et al., 2021). For example, in liver cancer (Yu and Schwabe, 2017; Ma et al., 2018; Zhang et al., 2021a) and breast cancer (Kwa et al., 2016; Chen et al., 2019), it has been confirmed that the transformation of primary bile acids into secondary bile acids by microorganisms may cause DNA damage, hepatotoxicity, change of NK T cell concentration and carcinogenesis. In breast cancer, gut bacteria lead to human steroid metabolism disorder, making estrogen content and distribution changes (Kwa et al., 2016; Xue et al., 2018). Thus, the investigation of gut microbiota associated with cancer progression gives much insight into related clinical applications. However, studies on the gut flora of cervical cancer are quite limited so far.

This study collected fecal samples from cervical cancer patients and normal controls to compare the different microbial signatures. In addition, the changes in gut microflora and their corresponding functions were also studied. Compared with the healthy control group, the results showed significant differences in gut microflora classification composition and diversity in patients with cervical cancer. Although α-diversity did not show a difference in the abundance of gut flora, β-diversity indicated the difference between the CC group and HCs group. The composition of the gut microbiota of CC and HCs was dominated by *Firmicutes*, *Bacteroidetes*, *Proteobacteria*, and *Actinobacteria*. However, their abundance was significantly different between CC and HCs groups. BMI was negatively correlated with *Bacteroides Plebeius*. Disease and stage were significantly and negatively correlated with *Ruminococcus 2*. And stage showed a positive correlation with *Escherichia* Shigella. Interestingly, we found that *Ruminococcus 2* was most closely related to cervical cancer in the gut flora family. These findings provide preliminary evidence that those with cervical cancer have distinctive gut flora.

Numerous research has supported the association between gut microbiota and non-digestive disorders (Kwa et al., 2016; Mohajeri et al., 2018; Ochoa-Repáraz et al., 2018; Peirce and Alviña, 2019). However, most studies are based on low-throughput methods or cultures of microbial flora, such as real-time fluorescence PCR and 16S rRNA amplification denaturing gradient gel electrophoresis. These findings only represent a small portion of the whole microbe. As a result, it is still very difficult to comprehend the general state of the gut microbial population and the accompanying microbial ecology and function. Since there is a lot of sequence variability among different bacteria, 16S rRNA genes of entire gut bacteria were chosen for sequencing in this work. These genes were typically grouped into 9 “hypervariable regions” (V1-V-9). Sequences that discriminate between a small number of distinct bacterial species or that identify particular bacterial species have been isolated through studies. In addition to 16S rRNA sequencing analysis, mNGS testing was added. The mNGS testing involves sequencing millions of DNA fragments in parallel (Behjati and Tarpey, 2013; Han et al., 2019). Piecing together individual reading fragments by mapping them to the reference genome. Each piece of DNA is sequenced multiple times, providing greater depth for accurate data and the discovery of different species. Therefore, this experiment has a better understanding than 16S rRNA sequencing analysis alone. In addition to diversity, the functions of some bacteria and their corresponding differential metabolic pathways were revealed.

In this study, the dominant expression of estrogen metabolism-related bacteria, such as *Bacteroides*, was also found in the dominant bacteria (Flint et al., 2012). Cervical cancer’s onset and progression may be linked to estrogen metabolism-related bacteria. A previous study showed that estrogen helps cervical cancer using a mouse model (Elson et al., 2000). The transformation region of transgenic HPV16 mice was 5 times more sensitive to estrogen-induced squamous cell carcinogenesis compared to the rest of the reproductive tract. In addition, another study also reported higher cervical cancer analysis in women with the highest circulating estrogen levels (Chen et al., 2021; Wang et al., 2021). We also found that *Ruminococcus 2* was most closely related to cervical cancer in the gut flora family, which could be considered a potentially relevant biomarker for the prediction of cervical cancer development. It has been found that *Firmicutes*, especially *Ruminococcus*, played an important role in polysaccharide degradation (Setchell and Clerici, 2010). *Ruminococcus* plays an important role in human metabolism by converting cellulose into various nutrients of the host. *Ruminococcus* is also closely related to the intestinal barrier, cellular immunity, inflammation, and metabolism (Hall et al., 2017; Schluter et al., 2020; Rangarajan et al., 2022). A recent study found that the absolute abundance of *Ruminococcus 2* showed a positive correlation with the number of lymphocytes (Schluter et al., 2020). The human immune effect is enhanced when the abundance of Ruminococcus 2 is higher. However, whether *Ruminococcus 2* affects the occurrence and development of cervical cancer in the host requires further biofunctional studies (Goedert et al., 2015; van der Meulen et al., 2016).

The major shortcoming of this study is the number of specimens collected. Therefore, we cannot clearly distinguish whether different stages and precancerous lesions have an impact on the gut microflora. In addition to HPV, other risk factors, such as multiple sexual partners and different living environments, were not analyzed. At the same time, the correlation between different flora and metabolic pathways associated with the occurrence and development of cervical cancer cannot be clarified without further functional experiments. Further prospective studies of longitudinal sampling of the microbiota of patients with persistent HPV-positive precancerous lesions are needed to determine whether progressive disruption of the microbiota contributes to cervical cancer.

## 5 Conclusion

Cervical cancer patients have different gut microbiome diversity compared with healthy women. This difference is reflected both in terms of community structure, as defined by increased or decreased diversity of the gut microbiome, and in terms of functional composition. The presence of microbial composition and specific functional pathways that may be necessary for the development of cervical cancer have been compared using prospective data.

## Data availability statement

The data presented in the study are deposited in the Genome Sequence Archive (Genomics, Proteomics & Bioinformatics 2021) at the National Genomics Data Center (Nucleic Acids Res 2022), China National Center for Bioinformation/Beijing Institute of Genomics, Chinese Academy of Sciences. The accession number is CRA009631.

## Ethics statement

The studies involving human participants were reviewed and approved by the First Affiliated Hospital of Zhengzhou University. The patients/participants provided their written informed consent to participate in this study. Written informed consent was obtained from the individual(s) for the publication of any potentially identifiable images or data included in this article.

## Author contributions

Conceptualization: NL, RG, and LC. Funding acquisition: NL and LC. Project administration: LC. Supervision: LC. Validation: LQ, JZ, SD, MC, FW, and BQ. Visualization: LQ, JZ, SD, MC, and FW, and BQ. Writing-original draft: LC, LQ, and NL. Writing-review and editing: NL and LC. This work was carried out in collaboration among all authors. All authors contributed to the article and approved the submitted version.
